# Endophtalmie tardive compliquant une chirurgie filtrante: à propos d'un cas

**DOI:** 10.11604/pamj.2015.20.432.6710

**Published:** 2015-04-30

**Authors:** Adil Bouzidi, Said Iferkhass

**Affiliations:** 1 Hopital Militaire Moulay Ismail, Meknes, Maroc

**Keywords:** Endophtalmie tardive, glaucome, hyperhémie conjonctivale diffuse, liquide fibrino-purulent, late endophthalmitis, glaucoma, diffuse conjunctival hyperemia, suppurative exudate

## Image en medicine

Bien que l'endophtalmie bacterienne est rare après chirurgie du glaucome, qui est aujourd'hui de plus en plus recommandée par de nombreux auteurs, sa parfaite connaissance par le chirurgien est indispensable. Si l'infection peut se déclarer de façon précoce après la chirurgie avec une présentation similaire à celle d'autres chirurgies réglées, à distance, il existe également des cas d'endophtalmie tardive survenant des mois, voire des années, après une période postopératoire calme. Nous rapportons le cas d'une patiente âgée de 62 ans, suivie pour glaucome primitif à angle ouvert depuis 2006, opéré de trabeculectomie en octobre 2007. Après une période postopératoire de un an et demi parfaitement calme, la patiente a présenté un tableau de conjonctivite bactérienne avec blébite traitée par antibiothérapie locale et qui s'est compliqué une semaine après par une endophtamie avec à l'examen clinique: hyperhémie conjonctivale diffuse, liquide fibrino'purulent à l'intérieur de la bulle de filtration et une fente purulente intra'oculaire. Juste après un prélèvement conjonctival, nous avons démarré une antibiothérapie systémique (ciprofloxacine et imedipime) suivie 24 heures après d'une injection d'antibiothérapie en intravitréenne-de la vancomycine à la dose de 1 mg et de la ceftazidime à la dose de 2 mg, renouvelée 48heures après. Le germe retrouvé au prélèvement conjonctival était Staphylococcus aureus. Une nette amélioration clinique a été obtenue après traitement sur le plan anatomique mais sur le plan fonctionnel l'acuité visuelle restait limitée à une perception lumineuse positive. Cette acuité visuelle s'expliquait par la persistance d'une gangue fibreuse au pôle postérieur.

**Figure 1:**
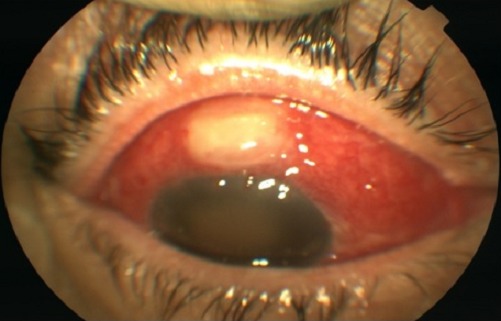
Blébite avec endophtalmie OD

